# Urethral Prolapse: Contemporary Report on a Modified Ligation Over a Urethral Catheter Treatment Approach

**DOI:** 10.5812/numonthly.11012

**Published:** 2013-07-23

**Authors:** Chukwudi Ogonnaya Okorie

**Affiliations:** 1 Department of Surgery, Banso Baptist Hospital, Kumbo, Cameroon; 2 Department of Surgery, Tenwek Hospital, Bomet, Kenya

**Keywords:** Catheters, Ligation, Prolapse, Urethra

## Abstract

**Background:**

Most contemporary series on urethral prolapse report either on the use of excisional or conservative treatment approaches.

**Objectives:**

To introduce a modified ligation over a Foley catheter treatment method for urethral prolapse that addresses most of the previously reported complications.

**Patients and Methods:**

Five consecutive patients with urethral prolapse treated between 2003 and 2011, all using the ligation method on an outpatient basis were studied prospectively. Maintaining the inflated balloon of the Foley catheter with timed removal of the catheters among other modifications to the original technique is further described in the article. The main outcome measures were to evaluate for recurrence, post-operative appearance of the urethral orifice and satisfaction of parents. Secondarily the actions of the parents of the patients and those of the receiving physicians were also recorded.

**Results:**

The mean age of the patients was 6 years old (ranging from 3 to 8 years). All parents suspected sexual molestation and in two cases, the suspected perpetrators were verbally threatened of dire consequences of their actions if proven. None of the receiving medical personnel were aware of this condition. Maximum length of catheterization was for 4 days. The post treatment urethral openings appeared normal and there were no complications.

**Conclusions:**

The ligation method with attention to the modifications described further in the article is a simple, safe and cost effective option for the management of urethral prolapse.

Maintaining the inflated balloon of the Foley catheter with timed catheter removal especially adds predictability to this technique.

## 1. Background

Urethral prolapse is a condition characterized by protrusion of the urethral mucosa beyond the external urethral meatus; the cardinal sign, usually noted on the underwear by the child or her parent, is scant to moderate bleeding from the urogenital area. This condition is very rare and mostly affects pre-pubertal black girls (1, 2) but has also been reported for other races (3-6). Probably due to its rare occurrence, urethral prolapse is still largely unknown and easily misdiagnosed (7).

The treatment options for urethral prolapse probably due to its rarity have remained variable and controversial, ranging from conservative or non-operative approaches to various operative interventions (1, 2, 8-10). In recent times, the debate on the appropriate treatment approach focuses predominantly on two treatment modalities: the conservative (non-operative) and the excisional variant of the operative method (2, 8-11) with other treatment modalities receiving less attention. The ligation over the urethral catheter is an old treatment approach (12, 13) that has had variable outcomes in the hands of different authors and not withstanding its numerous strengths and simplicity, it has been less frequently reported in recent years. The original method entails the use of either non-self retaining urethral catheter or use of self-retaining catheter with subsequently deflated balloon for later spontaneous catheter fall out following sloughing of the ligated tissue (12, 13). Many authors have reported very good outcomes for the ligation method (12-14). However some other authors have reported complications that include; premature discharge of the catheter, long catheterization, incomplete sloughing of the prolapsed urethral mucosa, urethral catheter obstruction, infection and post-operative pain and do not recommend its use (1, 6).

## 2. Objectives

The main aim of this study was to report on the contemporary use of an improved ligation method in the management of urethral prolapse, highlighting modifications made to avoid previously reported complications.

## 3. Patients and Methods

This was a prospective collection of data of five patients with urethral prolapse treated surgically between January 2003 and October 2011 on an out-patient basis at the departments of surgery of Banso Baptist hospital cameroon and Tenwek hospital Kenya. Pre-operative evaluation consisted of a detailed history of the disease and included questions on onset of illness, duration, presence of pain and urinary symptoms. Furthermore the children and the parents were allowed to freely narrate what they felt caused the bleeding and the actions they took to resolve the situation. All patients had focused preoperative physical examinations of the perineum and urinalysis.

**Figure 1.**
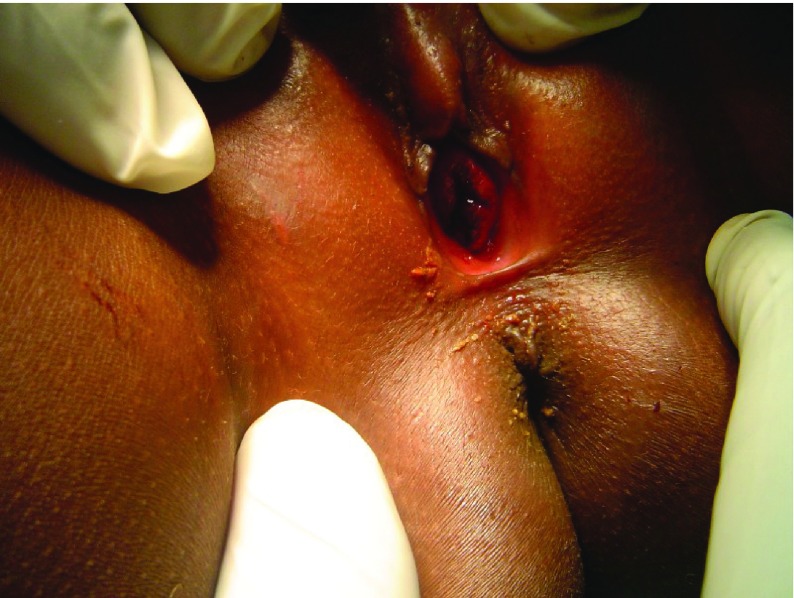
Urethral Prolapse

**Figure 2.**
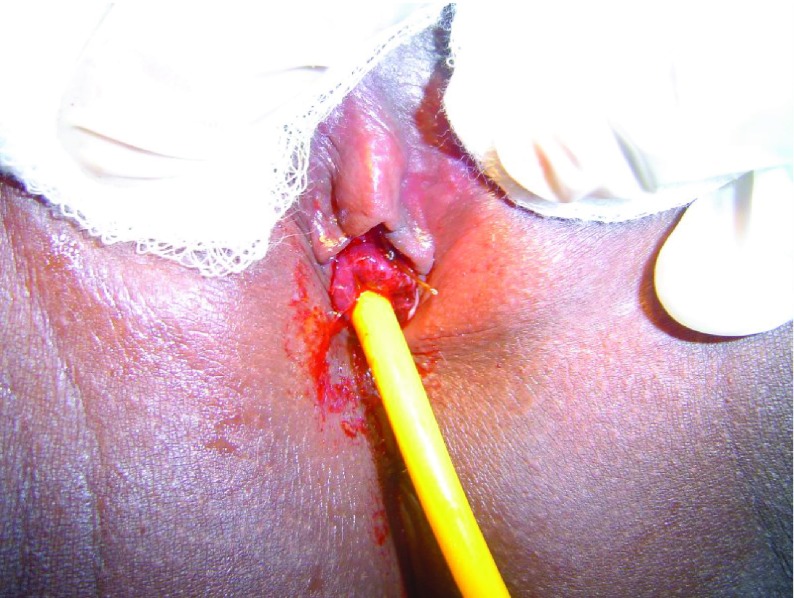
Urethral Prolapse Ligated Over a Foley Catheter

Confirmation of the diagnosis of urethral prolapse was made by observing a circular hemorrhagic mass, anterior to the vaginal orifice, covering the normal location of the urethra but with a central opening (Figure 1). Patients were treated surgically by the method of ligation over a Foley catheter under sedation and analgesia (Figure 2); The inserted and inflated Foley catheter (size 14 for all patients except usage of size 16 for one 8 year old patient) should be placed on mild traction and maintained as such by an assistant during ligation of the mass to ensure that the base of the mass is completely exposed, facilitating placement of the knot of the ligature (size 0 polyglactin suture material in this series) at the base of the mass. During the ligature, the following modifications where incorporated specifically to address previously reported complications: 

1. To avoid premature falling out of the urethral catheter: the practice, apart from the first two cases was not to deflate the balloon of the Foley catheter contrary to the original descriptions of this method.

2. To avoid long catheterization resulting from unpredictable time of spontaneous catheter fall out; timed and scheduled manual catheter removal was implemented.

3. For effective and complete sloughing of the prolapsed urethral mucosa: the ligature at the base of the prolapsed mass should be tied down to a strangulating depression at the demarcating line- a gentle and maintained pull on the catheter exposes the base hence showing the demarcation between the prolapsed mass and the proximal normal urethral tissue.

4. To avoid partial sloughing: all prolapsed tissues should be confirmed as distal to the ligature by circumferential inspection during ligature placement.

5. To prevent obstruction from the ligature: a reasonably large catheter size should be used and the patency of catheter lumen can be immediately tested by freely flushing normal saline solution through the lumen with a 60cc syringe without any noticeable resistance as well as observing backflow of the normal saline solution or urine out of the catheter during application of the ligature.

6. To prevent infection, prophylactic antibiotics coverage and a shorter period of catheterization should take place.

The Catheter is strapped to the thigh and connected to a urine bag. A course of oral antibiotics was given for one week to all patients starting from the day of surgery to 3 days after Foley catheter removal along with oral analgesics. In the first two patients the catheter balloon was deflated for spontaneous catheter discharge while the remaining patients were scheduled for manual catheter removal on post-operative day 4 from surgery. Post-operatively, patients were seen in the clinic one week from the procedure, then at one, three and twelve month’s post-operatively specifically to assess the urethral opening for recurrence and to obtain urine analysis for presence of infection. Parents of the patients were also given the cell phone number of the author to call for any problems.

## 4. Results

**Figure 3.**
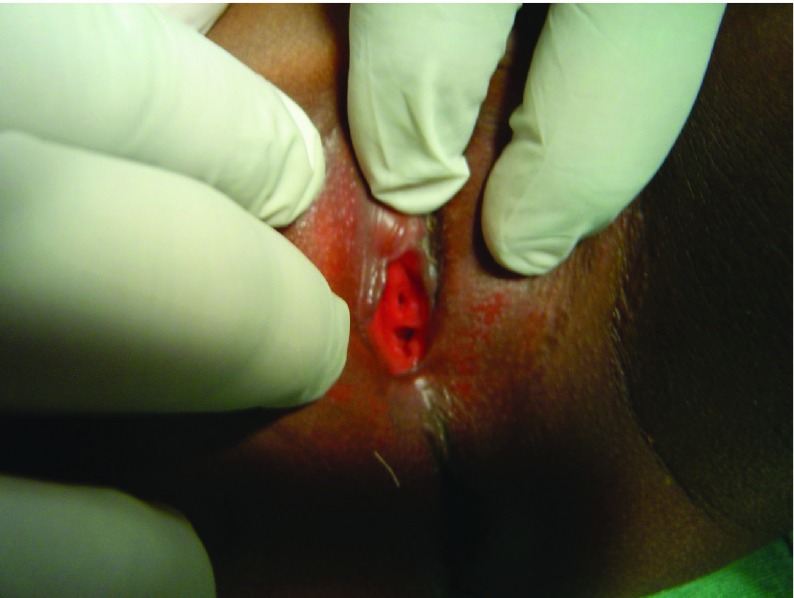
Urethral Opening After Treatment

The mean age of the patients was 6 years (ranging from 3 to 8 years). The chief complaint for all of the patients was the presence of blood stains on their underwear. All pre-operative urinalysis was negative for infection. For all cases, the involved parents suspected sexual molestation as the cause of the bleeding; in two instances, they had already verbally confronted and threatened these suspected persons in their communities of habitation of further dire consequences of their actions if proven. Prior to secondary assessment by the consultant, receiving medical personnel had no awareness of the urethral prolapse as a possible cause of the observed bleeding. For the first two patients in this series, the catheters fell off together with the ligated tissues three and a half days and four days post-operatively. The scheduled removal of the catheters together with the remnant of the necrotized tissue for the rest of the patients was easily achieved on post surgery day 4. On follow up, patients had no complaints and tolerated the treatment very well, the urethral opening appeared normal (Figure 3) and there was no infection in the control urine analysis. The parents were satisfied with the treatment and the post-operative appearance of the urethral orifice. Those parents that had confronted and threatened suspected members of their community, on knowing the correct diagnosis were able to apologize and reconcile with these suspected persons. 

## 5. Discussion

In this study, 5 patients with urethral prolapse were treated with a good outcome. The treatment options for urethral prolapse remain controversial. However two dominant treatment approaches have emerged over the recent years: the medical (conservative) method that includes sitz baths, topical antimicrobials, topical estrogen creams (1, 9-11) and the excisional operative approach (1, 2, 8). The acceptability of any of these treatment options remains controversial and variable. Some of the problems associated with the conservative approach include; (8) long persisting prolapse (7, 9, 11); significantly higher failure rate, Jerkins et al. (15) reported recurrence of up to 67% after conservative treatment; persistent bloody spotting with associated discomfort (8); theliarchy development with topical estrogen (11); high treatment cost, Hillyer et al. (8) estimates much higher treatment cost compared to the excisional method because of long and multiple clinic follow-up. Some of the drawbacks associated with the surgical excision of the prolapsed mucosa include: more invasiveness, high surgical cost (8), need for in-patient admission and general anesthesia (3, 6, 8), post-operative bleeding (4, 13) – Owens and Morse (13) reported a case of significant bleeding that necessitated blood transfusion with the excisional method, urethral stenosis (4), recurrence after excision (2, 4). In fact some authors feel that surgical excision does not have a place in the initial management of this condition (10, 16) while on the other hand, other authors see the excisional method as prompt, safe, more effective and being required anyway, in most cases due to the high failure rate of the conservative method and as such should be the primary approach (2, 5, 8).

In this series, a uniformly successful and complication free treatment with an improved ligation method has been possible. This approach was chosen as a less invasive, relatively quicker and cheaper treatment option with less extensive follow-up protocol. It is a simple procedure which if properly done allows for cost-effective and outpatient management of these patients and certainly can be especially beneficial for patients in low-resource economies as it is not technically demanding and doesn’t use up much supplies. The average surgical cost in this series in the study locations was 23.4 USA dollars.

Modifications of the original technique incorporated in this series did play a role in avoiding previously reported complications. Of particular interest is the frequently reported premature fall out of the urethral catheter before sloughing of the ligated tissue (1, 17). The key component of the original ligation method involving the use of non-indwelling urethral catheter or subsequent deflation of the balloon of indwelling catheter after ligation to allow spontaneous discharge of the catheter possibly explains the frequently reported premature discharge of the catheter. Furthermore the time of spontaneous catheter discharge was unpredictable and could range from 1 to 8 days (6, 12). From authors’ earlier experience, the catheters commonly fall out around the 4th day from surgery and if not, from experience of other authors (13), it could be manually removed together with the necrotized tissue around the 4th post operative day. Hence for the later patients in this series, their catheters weren’t deflated but these patients were instead scheduled to report on the 4th post-operative day at which time the catheters were deflated and easily removed without any complication. Scheduling for timed catheter removal on post-operative day 4 (which is feasible as this study shows) will help prevent catheterization exceeding 4 days thus making this treatment approach more predictable. In this series size 14 and one size 16 catheters were used. Many other authors have not reported the sizes of the urethral catheters used and it can be expected that ligation of the prolapsed mass over catheters of small size could more likely cause catheter obstruction compared to the use of larger catheters. It was noted that the prolapsed urethra readily accepts catheters of larger sizes to patients’ age.

The main reason for reporting this small series is to introduce this modified ligation method and underscore the uniform good outcome obtained. Furthermore, we have highlighted the fact that involved parents not only uniformly assumed that the urogenital bleeding noted in their young pre-pubertal females resulted from sexual molestation but two of them went further to accost and threaten the suspected persons. This problem is confounded, especially in low resource settings where access to specialists is limited, if there is failure by first line medical personnel to recognize and accurately diagnose the condition and to appropriately reassure the child’s parents as they refer for proper treatment. Policing and child-protection agencies are also in need of increased awareness of this pathology as they are very often contacted either prior to or following medical assessment (18, 19).

This study is limited by the fact that the data are gathered from a single surgeon providing a non-randomized surgical treatment for a small number of patients. However, urethral prolapse is a very rare pathology and accruing large numbers will always be a challenge. In reporting this series, it is hoped that this modified ligation method with its good outcome will be attractive and more favorable for those that see the excisional method as excessively invasive for this pathology on one hand and on the other hand for those that see the conservative method as largely ineffective and secondarily assist in future development of clinical guidelines as more studies with larger series emerge.
